# IL-33 promotes gastric tumour growth in concert with activation and recruitment of inflammatory myeloid cells

**DOI:** 10.18632/oncotarget.28238

**Published:** 2022-06-01

**Authors:** Chau P. Tran, Michelle Scurr, Louise O’Connor, Jon N. Buzzelli, Garrett Z. Ng, Sharleen Chung Nien Chin, Lincon A. Stamp, Toshinari Minamoto, Andrew S. Giraud, Louise M. Judd, Philip Sutton, Trevelyan R. Menheniott

**Affiliations:** ^1^Tumour Immunology Group, Murdoch Children’s Research Institute, Melbourne, Victoria, Australia; ^2^Department of Paediatrics, University of Melbourne, Melbourne, Victoria, Australia; ^3^Mucosal Immunology Group, Murdoch Children’s Research Institute, Melbourne, Victoria, Australia; ^4^Department of Anatomy and Physiology, The University of Melbourne, Victoria, Australia; ^5^Division of Translational and Clinical Oncology, Cancer Research Institute, Kanazawa University, Kanazawa, Japan; ^*^Co-senior authors

**Keywords:** IL-33, gastric cancer, tumorigenesis, mast cells, macrophages

## Abstract

Interleukin-33 (IL-33) is an IL-1 family cytokine known to promote T-helper (Th) type 2 immune responses that are often deregulated in gastric cancer (GC). IL-33 is overexpressed in human gastric tumours suggesting a role in driving GC progression although a causal link has not been proven. Here, we investigated the impact of IL-33 genetic deficiency in the well-characterized *gp130*^F/F^ mouse model of GC. Expression of IL-33 (and it’s cognate receptor, ST2) was increased in human and mouse GC progression. IL-33 deficient *gp130*^F/F^ */Il33*^−/−^ mice had reduced gastric tumour growth and reduced recruitment of pro-tumorigenic myeloid cells including key mast cell subsets and type-2 (M2) macrophages. Cell sorting of gastric tumours revealed that IL-33 chiefly localized to gastric (tumour) epithelial cells and was absent from tumour-infiltrating immune cells (except modest IL-33 enrichment within CD11b^+^ CX3CR1^+^CD64^+^MHCII^+^ macrophages). By contrast, ST2 was absent from gastric epithelial cells and localized exclusively within the (non-macrophage) immune cell fraction together with mast cell markers, Mcpt1 and Mcpt2. Collectively, we show that IL-33 is required for gastric tumour growth and provide evidence of a likely mechanism by which gastric epithelial-derived IL-33 drives mobilization of tumour-promoting inflammatory myeloid cells.

## INTRODUCTION

Chronic inflammation is one of the major contributing factors to cancer predisposition [1]. In the stomach, gastritis induced by *Helicobacter pylori* infection is a recognized risk factor for the majority of gastric cancers (GC), and resolving gastritis by the eradication of *H. pylori* lowers the incidence of GC [2, 3]. Thus a better understanding of the inflammatory mediators of the tumour microenvironment that drive GC pathogenesis will be important for developing new therapeutic approaches to GC prevention and treatment.

Interleukin-33 (IL-33) is a member of the IL-1 family, recognized by cells expressing the cell surface receptor ST2, an IL-1 receptor family member [4–7]. The IL-33/ST2 axis drives production of T helper type 2 (Th2) associated cytokines through activation of nuclear factor kappa-light-chain-enhancer of activated B cells (NFκB) signaling. IL-33 is constitutively expressed by epithelial cells at mucosal barrier sites where it acts as an enhancer of Th2 and type 2 macrophage (M2) immune responses [4–7]. In the lung, epithelial cells have been shown to release IL-33 in response to tissue damage induced by inflammatory stress or viral infection, with the secretion of this cytokine contributing to the exacerbation of inflammation [8, 9]. IL-33 has been classified together with IL-1α and high mobility group protein B1 (HMGB1) as dual-function alarmins, which collectively act as signals to alert the immune system to the presence of tissue damage [10]. A recent study from our group reported that while IL-33 expression is down regulated in both humans and mice infected with *H. pylori* (and in the presence of associated gastritis), exogenous IL-33 causes gastric inflammation and metaplasia associated with a Th2 response, highlighting the complex role of IL-33 in promoting premalignant progression to GC.

The IL-33 receptor ST2 belongs to the IL-1 receptor family. The human ST2 gene encodes 4 isoforms, two of which are predominant: membrane bound ST2 and secreted soluble (s)ST2 [11, 12]. Soluble ST2, consisting of only the extracellular region of ST2, [13] acts as a non-signaling decoy receptor that, via sequestration of IL-33 ligand, functions as a negative regulator of IL-33 signaling [14]. Membrane bound ST2 is expressed on a variety of immune cells including Th2 lymphocytes, mast cells, basophils, eosinophils, macrophages, type 2 innate lymphoid cells (ILC2) and natural killer (NK) cells, [15–21] whereas sST2 is produced by epithelial cells and fibroblasts [22].

The IL-33/ST2 axis has been shown to drive progression of several solid malignancies. High levels of IL-33 expression occur in gastric cancer, [23, 24] liver cancer, [25] colorectal cancer, [26] lung cancer, [27] breast cancer, [28] and are associated with poor outcome. In ovarian cancer, expression levels of both IL-33 and ST2 were negatively correlated with patient survival time, [29] while lower serum sST2 was found to be linked to malignant growth of colorectal cancer [30]. The authors reported that in xenograft models of metastatic colorectal cancer, sST2 was capable of inhibiting Th1 and Th2 responses, plus macrophage infiltration and polarization to type M2a. On the other hand, exogenous IL-33 expression in aggressive melanoma and mammary tumour cell lines was found to inhibit xenograft tumour growth through infiltration of CD8^+^ T cells and NK cells [31]. As such, IL-33 appears capable of either promoting inflammation leading to tumorigenesis or altering anti-tumour immune responses, possibly depending on which immune cells receive IL-33/ST2 signaling.

A recent analysis of ST2 genetic deficiency in a GC mouse model highlighted the importance of IL33/ST2 signaling in activating mast cells, which secrete chemotactic cytokines leading to the accumulation of pro-tumorigenic macrophages that drive gastric cancer progression [32]. Though revealing a requirement for ST2 in this process, there was no direct evidence supporting a functional role for the IL-33 ligand itself. Here, using IL33 ligand genetic deficiency we affirm the role of IL33 signaling in GC progression via IL-33, and additionally provide evidence that unidirectional signaling by gastric (and tumour) epithelium-derived IL-33 via ST2 on mast cells is a critical component of gastric tumour progression.

## RESULTS

### Increased IL-33 and ST2 expression in human and mouse GC

**Figure 1:**
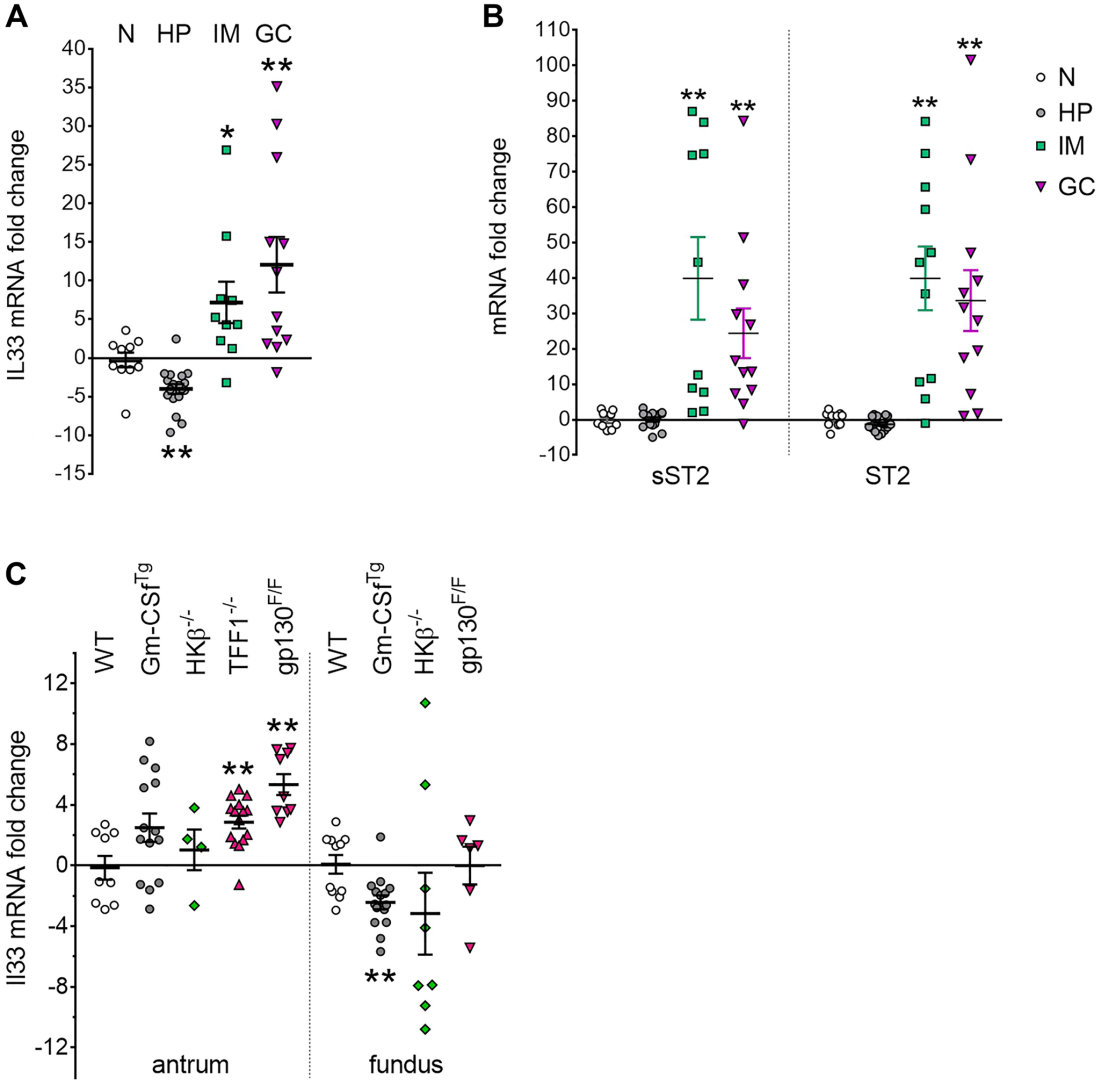
IL33 and ST2 overexpression in human and mouse gastric cancer. (**A**, **B**) qRT-PCR analysis of (A) *IL33*, (B) *sST2* and *ST2* expression in human gastric tissues. Graphs displayed mRNA fold changes in H. pylori infected gastritis (HP) (*n* = 18), preneoplastic adjacent tissue showing intestinal metaplasia (IM) (*n* = 10), and intestinal-type gastric cancer (GC) (*n* = 12) compared to normal stomach (N) (*n* = 12). (**C**) qRT-PCR analysis of *Il33* in genetic mouse models of gastritis, metaplasia and gastric cancer, showing mRNA fold change in the antrum and fundus compared to wild-type (WT) group. Two-tailed Student’s *t* test: ^*^ *P* < 0.05; ^**^ *P* < 0.01.

While high levels of IL-33 have been reported in sera and tissues of GC patients compared to normal controls [23], there is little information on comparative IL-33 expression in pre-cancer to GC development. Therefore, *IL33* gene expression levels were analyzed by quantitative (q)RT-PCR in human gastric tissues from *H. pylori* positive subjects exhibiting gastritis (HP), pre-neoplastic adjacent-to-cancer intestinal metaplasia (IM) and gastric cancer (GC) as well as disease-free normal controls (N). These comparisons showed a decrease in *IL-33* expression in the HP group compared to normal controls (−4.02 ± 0.64; *P* < 0.01; Figure 1A). However, a significant fold increase in *IL33* mRNA expression was detected in both the IM (7.18 ± 2.69, *P* < 0.05) and GC groups (12.05 ± 3.59, *P* < 0.01), compared to the control group. No significant difference was seen between the IM and GC groups. Analysis of the IL-33 receptor ST2 revealed no change in the HP group, but significant fold increase in the IM (39.87 ± 8.96, *P* < 0.01) and GC (33.61 ± 8.57, *P* < 0.01) groups. A similar pattern of expression was also seen with the decoy receptor, sST2 (Figure 1B). his suggests a pro-tumorigenic role for the IL-33/ST2 axis in early, as well as in later stage GC progression.

Altered *Il33* expression was similarly observed in selected mouse gastric tumorigenesis models (Figure 1C). *ll33* expression increased significantly (5.33 ± 0.69, *P* < 0.01) in the tumour-bearing antrum of gp130^F/F^ mice, which show fully penetrant GC by 12 weeks of age, [33] remaining unchanged in the tumour-free fundus. A similar pattern of increased *Il33* expression (2.86 ± 0.43, *P* < 0.01) was seen in *Tff1*^−/−^ mice (Figure 1C), which develop severe antral hyperplasia and subsequent GC at 20 weeks of age [34].

### IL33 dependent gastric tumorigenesis in gp130^F/F^ mice

**Figure 2:**
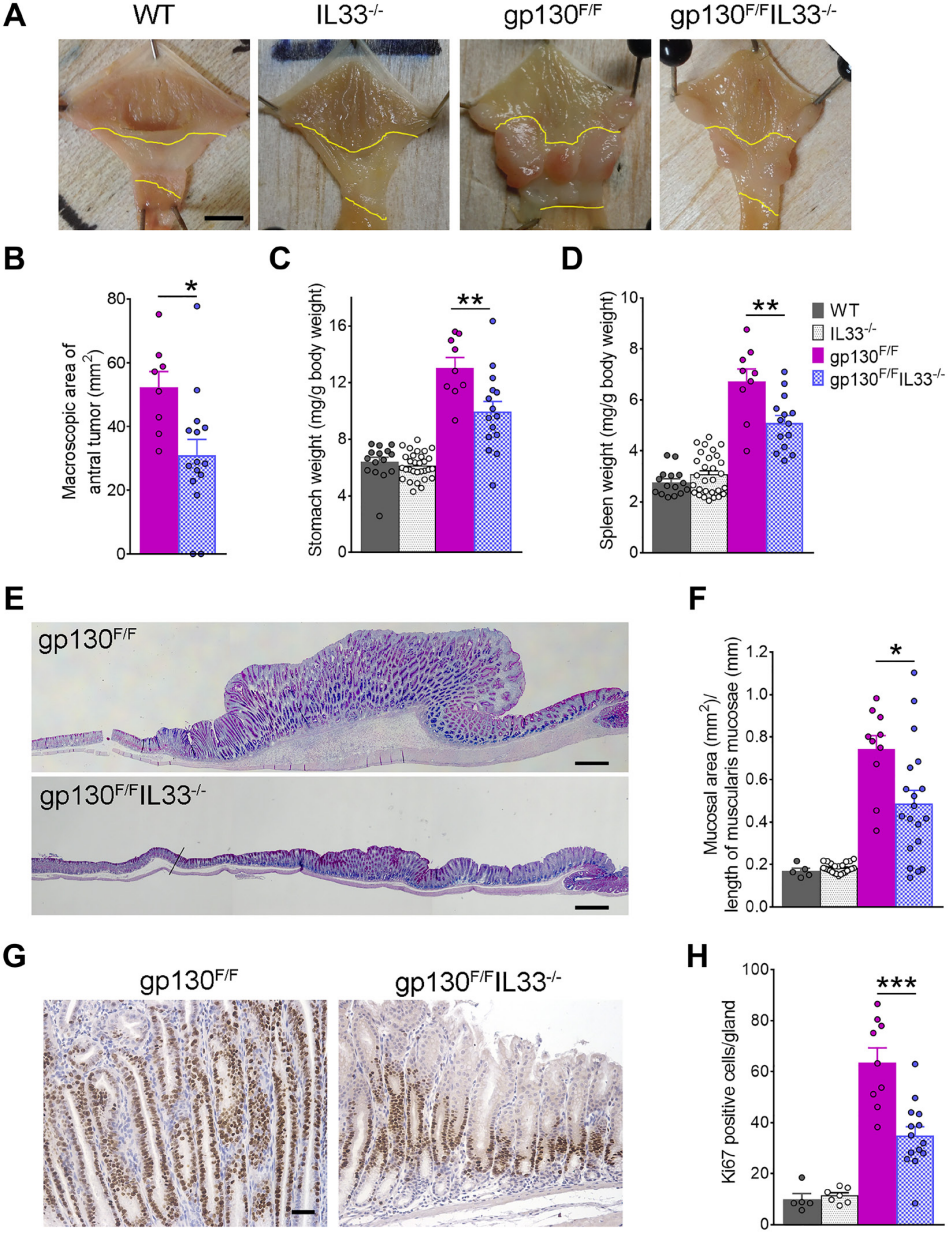
Gastric tumorigenesis in *gp130*^F/F^ and *gp130*^F/F^IL33^−/−^ mice. (**A**) Representative images of 12 weeks old *gp130*^F/F^ and *gp130*^F/F^/*Il33*^−/−^ stomachs, showing difference in antral tumour sizes compared to tumour-free stomachs of *Il33*^−/−^ and WT mice. Antral areas are outlined between yellow lines. Scale bar: 5 mm. (**B**) Macroscopic morphometric analysis of antral tumours in *gp130*^F/F^ (*n* = 9) and *gp130*^F/F^/*Il33*^−/−^ (*n* = 15) mice. (**C**) Stomach and (**D**) spleen weight analysis of WT, *Il33*^−/−^, *gp130*^F/F^ and *gp130*^F/F^/*Il33*^−/−^ mice. (**E**) Representative histological images (AB-PAS staining) of the antral mucosa showing difference in tumour sizes. Scale bar: 0.5 mm. (**F**) Histological analysis of antral mucosal thickness in WT, *Il33*^−/−^, *gp130*^F/F^ and *gp130*^F/F^ */Il33*^−/−^ mice. (**G**) Representative images of Ki67 immunohistochemical staining of antral tumours. Scale bar: 100 μm. (**H**) Quantification of Ki67 staining as average number of Ki67 positive cells per gland in WT, *Il33*^−/−^, *gp130*^F/F^ and *gp130*^F/F^ */Il33*^−/−^ mice. Two tailed student’s *t* test: ^*^ *P* < 0.05; ^**^ *P* < 0.01, ^***^ *P* < 0.001.

As IL-33 is demonstrably overexpressed in both human and mouse GC, we asked whether loss of this cytokine might alleviate the effects of pathways know to induce gastric tumorigenesis in mice. A comparison of *IL33^−/−^* and WT mice revealed no difference in stomach and spleen weights, gastric mucosal thickness or cell proliferation (data not shown), showing that loss of IL-33 alone (i.e., in the absence of genetic or inflammatory drivers) does not significantly alter baseline gastric epithelial composition. Previous studies have identified the gp130/STAT3/IL-11 axis as a main driver of GC development [35, 36]. gp130^F/F^ mice, harboring the knock-in mutation of the gp130 co-receptor, show constitutive activation of gp130/STAT3 and develop gastric tumours in the antral stomach. This model recapitulates many steps of intestinal-type GC progression in humans, exhibiting chronic gastric inflammation, intestinal-type metaplasia, dysplasia and non-invasive cancer [33]. To determine the functional impact of IL-33 in gastric tumorigenesis, we generated and analyzed compound mutant *gp130*^F/F^/*Il33*^−/−^ mice. A comparison at 6 weeks of age revealed a small but significant reduction in stomach weight (8.24 ± 0.23 vs. 7.49 ± 0.30 mg/g body weight, *P* < 0.05), spleen weight (5.23 ± 0.23 vs. 4.63 ± 0.13 mg/g body weight, *P* < 0.05), and area of antral lesions (28.59 ± 1.42 vs. 20.54 ± 2.63 mm^2^, *P* < 0.05) in *gp130*^F/F^/*Il33*^−/−^ compared to *gp130*^F/F^ mice (Supplementary Figure 1A–1C). This implies loss of IL-33 might interfere with early tumorigenesis. At 12 weeks old, the stomachs of *Il33*^−/−^ mice were grossly normal with no visible lesions (as per the WT controls). In contrast, large tumours were observed in *gp130*^F/F^ antrum, compared to visibly smaller tumours seen in *gp130*^F/F^/*Il33*^−/−^ antrum (Figure 2A). Loss of IL-33 markedly reduced tumorigenesis in *gp130*^F/F^ antra, as shown by a significant decrease in macroscopic antral tumour area (52.23 ± 5.00 vs. 30.99 ± 4.94 mm^2^, *P* < 0.05) (Figure 2B). While the stomach weights of *gp130*^F/F^ mice were increased relative to WT and *Il33*^−/−^ mice, a comparison of *gp130*^F/F^ and *gp130*^F/F^/*Il33*^−/−^ stomach weights revealed a significant reduction (13.06 ± 0.72 vs. 9.94 ± 0.73 mg/g body weight respectively, *P* < 0.01) (Figure 2C). Splenomegaly, one of the pathologic phenotypes described previously in *gp130*^F/F^ mice, was also lessened in *gp130*^F/F^ *Il33*^−/−^ mice, as marked by a significant reduction of spleen weights (6.73 ± 0.48 vs. 5.10 ± 0.28 mg/g body weight respectively, *P* < 0.01) (Figure 2D). Histologically, in contrast to the poorly differentiated epithelia of *gp130*^F/F^ mice, compound mutant *gp130*^F/F^/*Il33*^−/−^ mice had significantly less antral thickening (0.74 ± 0.06 vs. 0.49 ± 0.06 mm, *P* < 0.05) and overall improved epithelial structure (Figure 2E, 2F). There were no differences in fundus mucosa between the two groups (Supplementary Figure 2A). Further evidence of reduced antral tumour growth in *gp130*^F/F^/*Il33*^−/−^ compared to *gp130*^F/F^ mice was seen in a reduction in the number of Ki67 positive cells in the gastric mucosa (63.7 ± 5.7 vs. 35.0 ± 3.5, *P* = 0.0002) (Figure 2G, 2H). Collectively these data identify IL-33 as a likely cell autonomous cytokine driver of tumour growth in GC.

### Reduced tumour-associated inflammation in IL-33 deficient gp130^F/F^ mice

**Figure 3:**
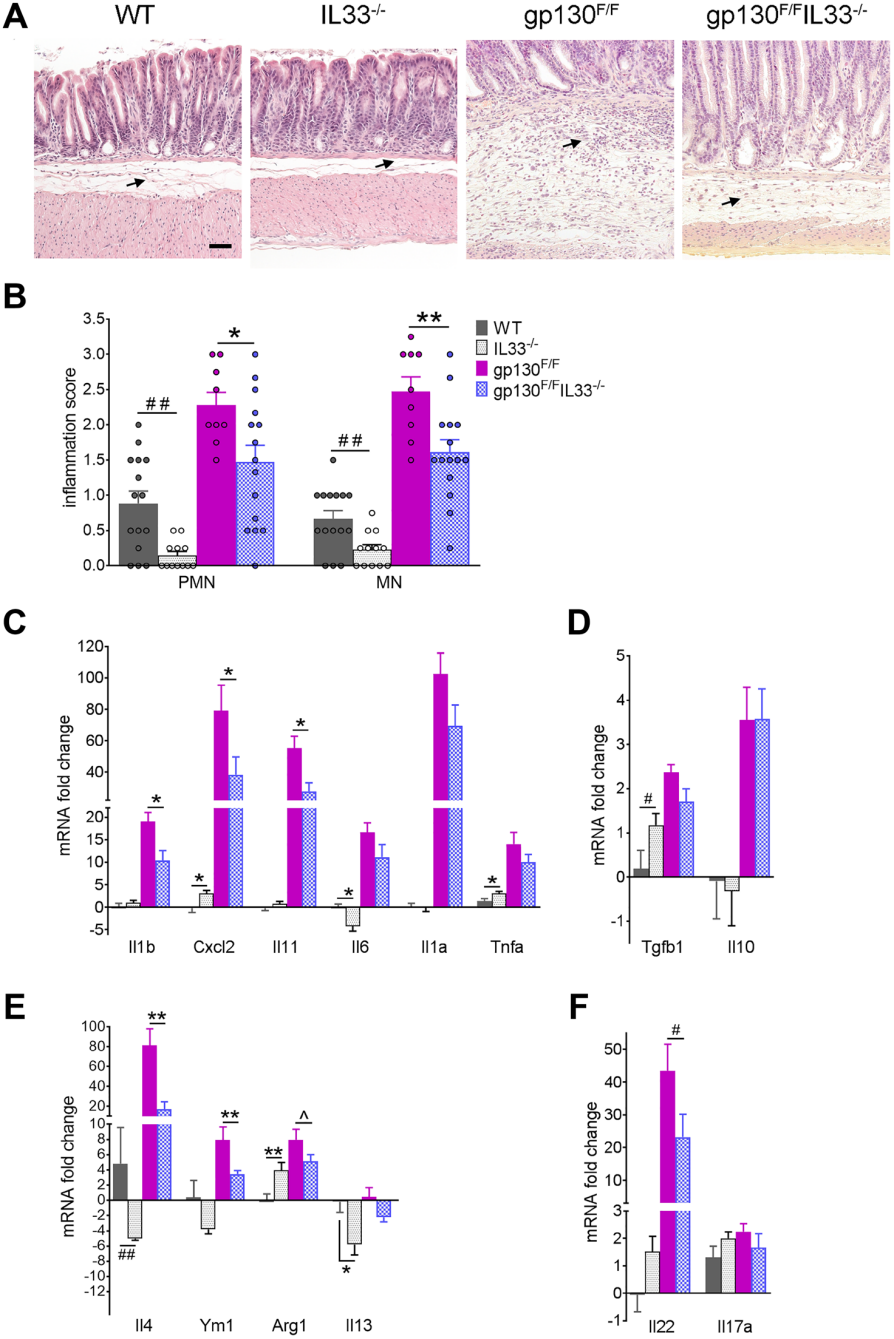
Gastric inflammation in gp130^F/F^ and gp130^F/F^IL33^−/−^ mice. (**A**) Representative histological images (H&E staining) of antral mucosa showing difference in inflammatory infiltrates in 12 weeks old WT (*n* = 15), *Il33*^−/−^ (*n* = 12), *gp130*^F/F^ (*n* = 9) and *gp130*^F/F^/*Il33*^−/−^ mice (*n* = 15). Arrows indicate inflammatory infiltrate. Scale bar: 100 μm. (**B**) Semiquantitative histological analysis of inflammation in the antral mucosa; PMN - polymorphonuclear cells, MN – mononuclear cells. (**C**–**F**) qRT-PCR analysis of antral tissues showing expression of (C) pro-inflammatory cytokines, (D) anti-inflammatory cytokines, (E) M2/Th2 markers, (F) Th22 and Th17 cytokines. Graphs showed mRNA fold change relative to WT. Two tailed Student’s *t* test: ^*^ *P* < 0.05, ^**^ *P* < 0.01. Mann Whitney test: ^#^ *P* < 0.05, ^##^ *P* < 0.01. One tailed Student’s *t* test: ^^^ *P* < 0.05.

We have reported previously that gastric inflammation drives tumour development in *gp130*^F/F^ mice [37]. We therefore asked if gastric inflammation in *gp130*^F/F^ mice was reduced with the loss of IL-33. Histological analysis of antral stomachs revealed a higher concentration of inflammatory infiltrate in the mucosal/submucosal space at the base of *gp130*^F/F^ tumours compared with the *gp130*^F/F^/*Il33*^−/−^ antrum (Figure 3A). Compared to those from *gp130*^F/F^ mice, tissues from *gp130*^F/F^/*Il33*^−/−^ mice showed a significantly lower infiltration of polymorphonuclear (PMN) (2.28 ± 0.19 vs. 1.48 ± 0.24, *P* < 0.05) and mononuclear (MN) (2.47 ± 0.21 vs. 1.61 ± 0.18, *P* < 0.01) cells (Figure 3B). Interestingly, whilst their gastric epithelial composition was effectively normal, *Il33*^−/−^ mice had a lower baseline gastric inflammation score than wildtype controls, in both antral (PMN 0.88 ± 0.18 vs. 0.15 ± 0.06, *P* < 0.01; MN 0.67 ± 0.12 vs. 0.23 ± 0.07, *P* < 0.01) and fundic (PMN 0.64 ± 0.08 vs. 0.28 ± 0.15, *P* < 0.01; MN 0.77 ± 0.07 vs. 0.44 ± 0.09, *P* < 0.01) mucosa (Figure 3A and Supplementary Figure 2B). This suggests that loss of IL-33 may lead to reduced recruitment of an IL-33-regulated immune cell population in the gastric mucosa, thereby removing a key inflammatory stimulus in the *gp130*^F/F^ antrum.

To better understand mechanisms underlying the reduced inflammatory infiltrate observed in the *gp130*^F/F^/*Il33*^−/−^ antrum, we first quantified by qRT-PCR expression levels of cytokines and chemokines known to be up-regulated in *gp130*^F/F^ antral tumours. These analyses showed that gastric tissues from *gp130*^F/F^/*Il33*^−/−^ mice expressed lower levels of proinflammatory *Il1b*, *Cxcl2* and *Il11*, compared to those present in *gp130*^F/F^ mice (Figure 3C). Since *gp130*^F/F^ tumour development has been shown to be driven by IL-11 upregulation, [35, 36] attenuated *Il11* expression and tumour growth seen in the compound mutant mice suggests that IL-33 might play a key role in the persistence of inflammation that leads to tumorigenesis. While the expression levels of *Il6*, *Il1a*, and *Tnfa* were also lower in *gp130*^F/F^/*Il33*^−/−^ compared to the elevated levels present in *gp130*^F/F^ mice, this difference did not reach significance. Similarly, expression levels of immunosuppressive *Tgfb1* and *Il10* were increased in *gp130*^F/F^ mice and remained elevated in compound mice (Figure 3D). Since IL-33 is a known enhancer of both Th2 immunity [4] and M2 macrophage polarization, [7] Th2/M2 associated markers were assessed by qRT-PCR. As expected, reduced expression of Th2-associated *Il4* and *Il13* was observed in *IL33^−/−^* compared to WT mice (Figure 3E). The antra of *gp130*^F/F^ mice expressed higher levels of *Il4*, *Ym1* and *Arg1*, which were significantly reduced in *gp130*^F/F^/*Il33*^−/−^ mice, while no change in *Il13* expression were seen in either group of *gp130*^F/F^ mice (Figure 3E). Analysis of Th17-associated cytokines (an important component of the pro-inflammatory response to *H. pylori* infection), revealed that *Il22* expression, which was highly elevated in *gp130*^F/F^ mice (compared to WT controls), was significantly reduced in the *gp130*^F/F^/*Il33*^−/−^ group, while there was no change in *Il17a* expression in any group (Figure 3F).

### IL33 dependent macrophage recruitment in gp130^F/F^ mice

**Figure 4:**
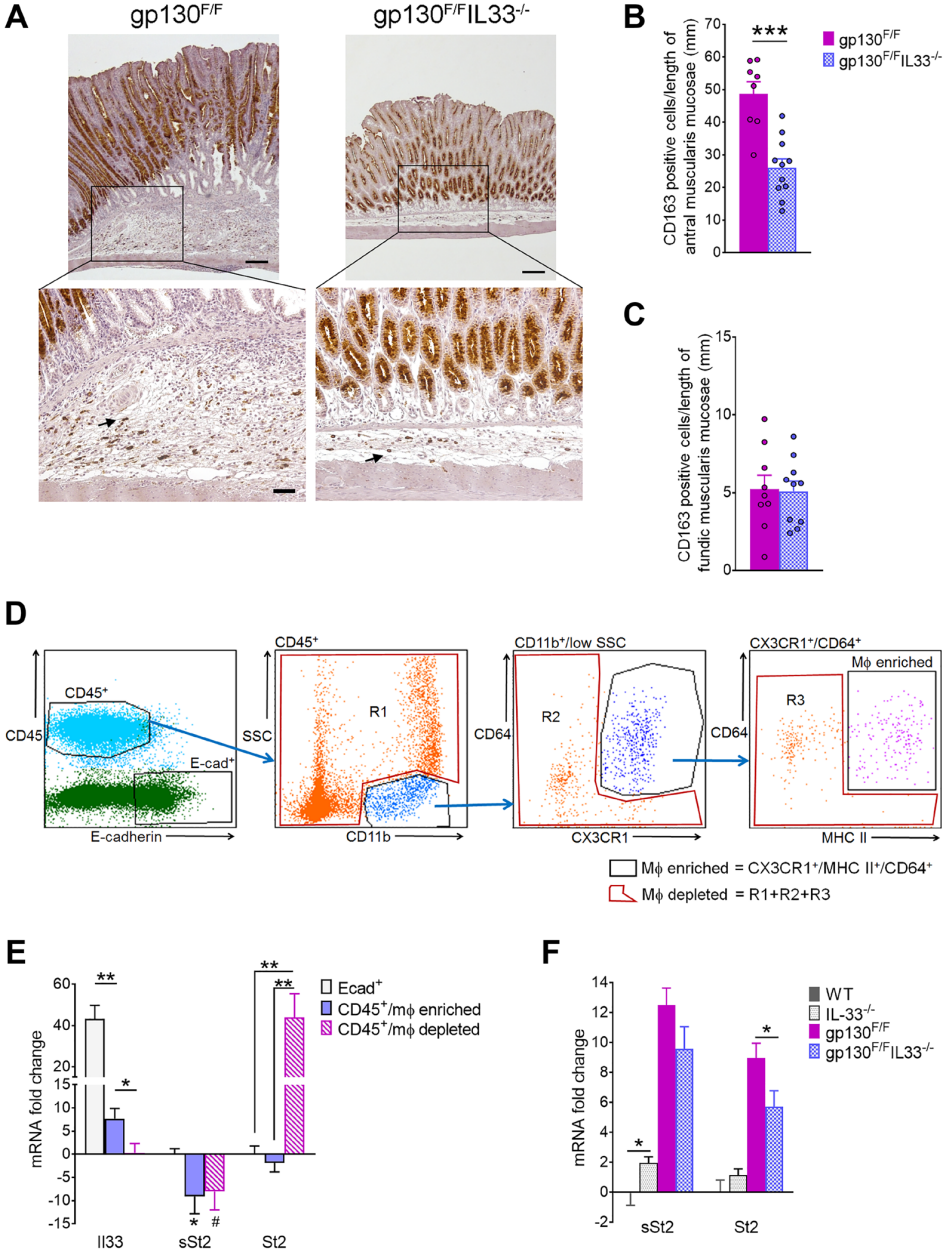
Molecular phenotyping of gastric inflammatory cells in 12 weeks old *gp130*^F/F^ and *gp130*^F/F^ *IL33*^−/−^ mice. (**A**) Representative images of CD163 immunohistochemical staining of antral tumours at ×40 magnification (upper panels, scale bar: 0.25 mm) and at ×200 magnification (lower panels, scale bar: 100 μm). Arrows indicate positively stained immune cells. (**B**, **C**) Quantification of CD163 staining as number of CD163 positive cells per length of (B) antral and (C) fundic muscularis mucosae. (**D**) Flow cytometry sorting of *gp130*^F/F^ gastric immune cells; gating strategy to isolate epithelial (CD45^−^/E-cad^+^), macrophage (Mφ) enriched (CD45^+^/CD11b^+^/SSC^low^/CD64^+^/CX3CR1^+^/MHCII^+^), and Mφ depleted (CD45^+^/Mφ depleted) populations. (**E**) qRT-PCR analysis of sorted gastric epithelial, Mφ enriched and Mφ depleted cell populations in *gp130*^F/F^ mice (*n* = 6). mRNA fold changes are calculated relative to Mφ depleted group for *Il33*, and relative to Ecad^+^ group for *sSt2* and *St2*. (**F**) qRT-PCR analysis of *sSt2* and *St2* in antral tissues from WT, *Il33*^−/−^, *gp130*^F/F^ and *gp130*^F/F^/*Il33*^−/−^ mice. Two tailed Student’s *t* test: ^*^ *P* < 0.05, ^**^ *P* < 0.01, ^***^ *P* < 0.001. Mann Whitney test: ^#^ *P* < 0.05.

We previously reported that IL-33 is specifically expressed by surface mucous cells of the gastric epithelium, and is capable of polarizing systemic macrophages to the M2 phenotype [7]. Furthermore, another previous study suggested macrophages as the major inflammatory cells in the *gp130*^F/F^ model [37]. To this end, we asked whether loss of IL-33 might affect macrophage accumulation at tumour sites. Immunohistochemical staining for the M2 marker CD163 revealed a high influx of CD163 positive cells to the base of *gp130*^F/F^ tumours, compared to a lower cell density in the antra of gp130^F/F^IL33^−/−^ mice (Figure 4A, lower left vs. right panel). Cell count analysis confirmed a significant reduction in the number of antral CD163 positive cells in *gp130*^F/F^/*Il33*^−/−^ compared to *gp130*^F/F^ mice (26.03 ± 2.71 vs. 48.7 ± 3.72 respectively, *P* < 0.001), while no difference was observed in the fundic tissues of these two groups (Figure 4B, 4C). Thus, IL-33 appears to promote increases in tumour-associated M2 macrophages in *gp130^F^*^/F^ mice.

Next, we assessed whether macrophages might contribute to the elevated *Il33* expression in *gp130*^F/F^ tumour, or if they might be recruited to tumour sites via IL-33 signaling. Tumours from *gp130*^F/F^ stomachs were dissected, disaggregated and sorted by flow cytometry into three populations: gastric epithelial cells (E-cadherin^+^/CD45^−^), macrophage (Mφ) enriched immune cells (CD45^+^/CD11b^+^/SSC^low^/CX3CR1^+^/CD64^+^/MHCII^+^) and Mφ depleted immune cells (CD45^+^/Mφ depleted) (Figure 4D). Purity of the sorted cell populations was independently validated by qRT-PCR showing expected enrichment of markers E-cadherin (*Cdh1*) and *Muc5ac* in E-cad^+^/CD45^−^ sorted gastric epithelial cells, and high *CD45* mRNA expression in both the Mφ enriched and depleted cell populations (Supplementary Figure 3A). Mφ enrichment was further confirmed by enrichment of Mφ marker gene expression (*Ccr2*, *Il4ra* and *S100A4* [38]) compared to the CD45^+^/Mφ depleted population (Supplementary Figure 3B). qRT-PCR analysis of all three cell populations revealed that whilst the highest *Il33* expression level was detected in epithelial cells, modest but significant IL-33 expression was also observed in the CD45^+^/Mφ enriched, but not the Mφ depleted population (Figure 4E). Expression of the sST2 isoform was depleted in both leukocyte populations compared to epithelial cells, consistent with previous report of sST2 expression in epithelial cells [22]. In contrast, all of the tumoral ST2 expression localized to the Mφ depleted/CD45+ cells, with ST2 otherwise absent from CD45^+^/Mφ enriched and gastric epithelial cell populations. qRT-PCR analysis of both receptor isoforms in antral tissues showed that both sST2 and ST2 were highly expressed in *gp130*^F/F^ mice, while only ST2 expression was significantly reduced in the *gp130*^F/F^/*Il33*^−/−^ group (Figure 4F), suggesting that IL-33 modulates the recruitment of ST2 expressing cells. Collectively, these data implicate the IL-33/ST2 signaling axis in promoting chronic and oncogenic inflammatory response that drives tumorigenesis.

### IL33 promotes recruitment of mucosal and connective tissue mast cell subsets in *gp130*^F/F^ mice

Immune subsets that constitutively express the IL-33 receptor, ST2, include Tregs, ILC2 and mast cells [39], are likely to be the primary mediators of IL-33 in the stomach. Mast cells in particular are noted among the most prominent of ST2 expressing cells (Supplementary Figure 4). A recent study established a role for ST2 in the recruitment of mast cells to *gp130*^F/F^ tumours but did not directly examine IL33 itself [32]. Thus, we next examined mast cells in IL-33 deficient *gp130*^F/F^ mice, focusing on the two major subsets, connective tissue mast cells (CTMC) and mucosal mast cells (MMC), which differ in their histochemical and molecular characteristics, cytokine profiles and crucially, role in tumour progression [40]. CTMC typically accumulate in submucosa at tumour margins and are detected by toluidine blue histochemical staining. In contrast, MMC typically localize *within* gastrointestinal epithelia (and tumours), are not toluidine blue reactive, being identified instead by selective expression of mucosal mast cell protease (mMCP) 1 and 2, encoded by *Mcpt1* and *Mcpt2* respectively [41, 42]. Recent work showed that activation of the MMC subset in particular mobilizes immunosuppressive and protumorigenic myeloid cells to drive gastrointestinal tumour growth [43].

**Figure 5:**
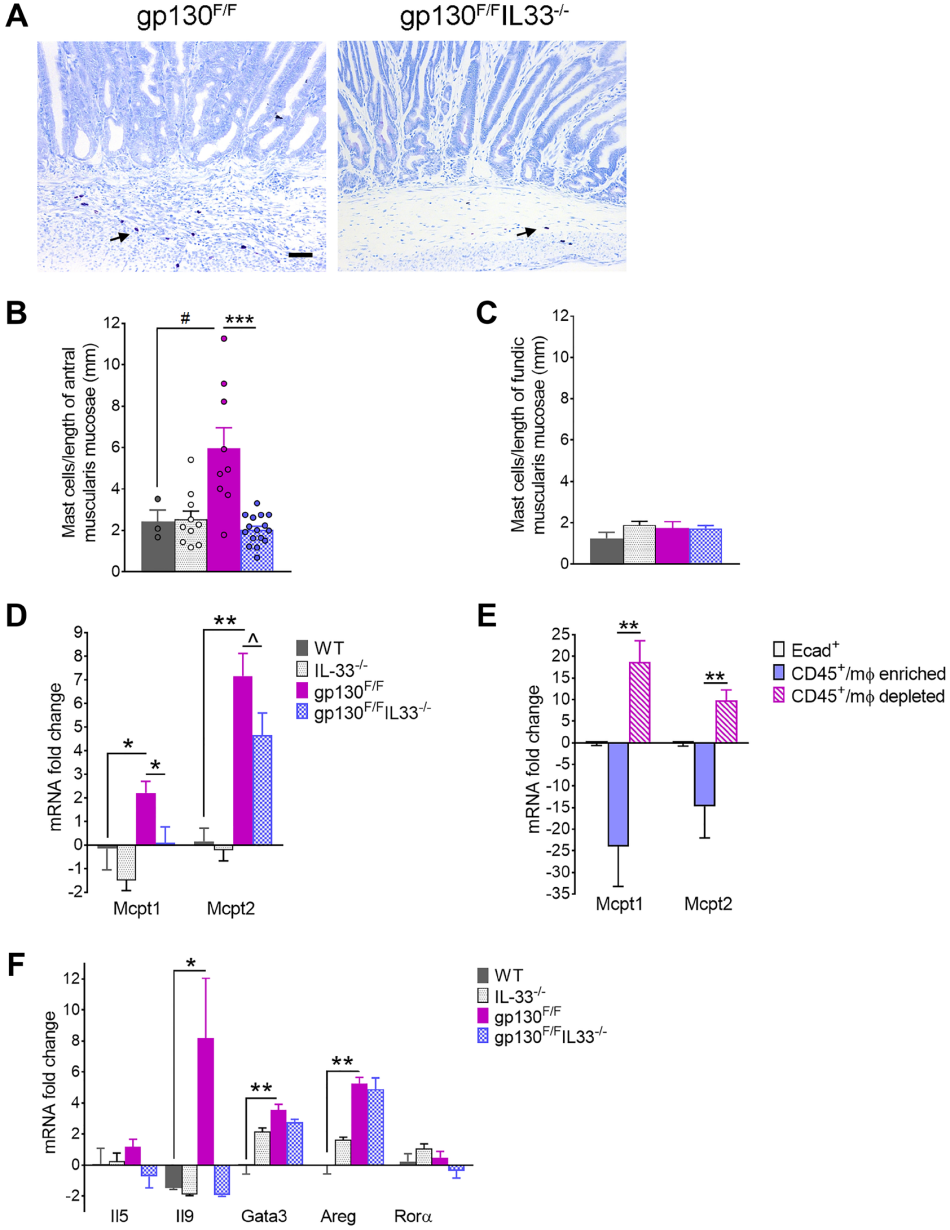
Mast cell profiling in 12 week-old *gp130*^F/F^ and *gp130*^F/F^/*Il33*^−/−^ mice. (**A**) Representative images of Toluidine Blue staining of antral tumours. Arrows indicate positively stained mast cells. Scale bar: 100 μm. (**B**, **C**) Quantification of mast cell staining as number of positive cells per length of (B) antral and (C) fundic muscularis mucosae. (**D**) qRT-PCR analysis of mast cell marker Mcpt1 and Mcpt2 in sorted gastric epithelial, Mφ enriched and Mφ depleted cell populations in *gp130*^F/F^ (*n* = 6). mRNA fold changes are calculated relative to Ecad^+^ group. (**E**, **F**) qRT-PCR analysis of antral tissues from WT, Il33^−/−^, *gp130*^F/F^ and *gp130*^F/F^/*Il33*^−/−^ mice showing expression of (E) mucosal mast cell markers and (F) group 2 innate lymphoid cells (ILC2) markers. Two tailed student’s *t* test: ^*^ *P* < 0.05; ^**^ *P* < 0.01, ^***^ *P* < 0.001. Mann Whitney test: ^#^ *P* < 0.05. One tailed Student’s *t* test: ^^^ *P* < 0.05.

Toluidine blue staining revealed increased accumulation of CTMC at the tumour margins and within the gastric submucosa in *gp130*^F/F^ compared to *gp130*^F/F^/*Il33*^−/−^ mice (Figure 5A). Cell counts confirmed a significant increase in numbers of CTMC infiltrating the antral stomach from *gp130*^F/F^, with counts comparatively attenuated in antra of *gp130*^F/F^/*Il33*^−/−^ compound mutants to levels similar to that of WT control mice (Figure 5A, 5B). IL-33 dependent mast cell accumulation was specific to antral stomach, with the fundic mucosa showing no difference in mast cell numbers between the experimental groups (Figure 5C). Similarly, quantification by qRT-PCR showed significantly increased expression of MMC markers, *Mcpt1* and *Mcpt2* in *gp130*^F/F^ antrum compared to WT controls, but was notably attenuated in *gp130*^F/F^/*Il33*^−/−^ antrum (Figure 5D). Localisation of MMC within tumours was indirectly confirmed by qRT-PCR analysis showing enrichment of *Mcpt1* and *Mcpt2* mRNA within the non-macrophage/CD45+ cell fraction isolated directly from *gp130*^F/F^ antral tumours by FACS (Figure 5E). These data suggest that in IL-33 deficient *gp130*^F/F^ mice, decreased prevalence of both CTMC and MMC subsets is correlated with reduced tumour growth.

To elucidate mechanisms by which IL-33 might induce mast cell expansion, we assessed markers of ILC2 cells, which as key immune mediators of IL-33 in the stomach were considered as potential upstream drivers of gastric tumorigenesis in *gp130*^F/F^ mice [7]. Consistent with activation of ILC2, qRT-PCR analysis of *gp130*^F/F^ antra showed increased expression of ILC2 markers *Gata3*, *Areg*, *Il9* and *Il4* (Figure 5F; Figure 3E), but importantly not all were induced in an IL-33 dependent manner, with *Gata3* and *Areg* increased similarly in antra and tumours of *gp130*^F/F^ and *gp130*^F/F^ *Il33*^−/−^ mice. By contrast, expression of *Il9* and *Il4,* known cytokine inducers of MMC and CTMC respectively [44, 45], showed a marked IL-33 dependent increase in *gp130*^F/F^ antral tumours, with levels in *gp130*^F/F^ *Il33*^−/−^compound mutants comparable to those of WT controls (Figure 5F; Figure 3E). As such, IL-33-dependent tumour growth in *gp130*^F/F^ mice correlated with the expansion of mast cell subsets but could not be definitively linked to ILC2, notwithstanding IL-9 (an ‘ILC2-related’ cytokine) as a putative mechanism for MMC expansion in this model. Collectively, these data identify mast cells as the primary immune mediators of IL-33 dependent gastric tumorigenesis.

## DISCUSSION

Here we demonstrate a direct functional role for IL-33 as a cytokine driver of gastric tumorigenesis. We show that IL-33 and its cognate signaling receptor, ST2, are overexpressed in human and mouse GC progression. In mouse genetic studies, we show that genetic ablation of IL-33 restrains tumour growth as well as limiting the recruitment of tumour promoting mast cells and immunosuppressive M2 macrophages, which are key drivers of gastric tumorigenesis. Reduced tumour burden in *gp130*^F/F^/*Il33*^−/−^ mice was correlated with decreased gastric epithelial proliferation, inflammation and protumorigenic cytokine/chemokine expression associated with alternative M2 macrophage activation. Our findings support a pivotal role for IL-33 as a gastric (and tumour) epithelium-derived “alarmin” that promotes a protumorigenic immune response, via unidirectional signaling through ST2 receptors on mast cells and via recruitment of immunosuppressive M2 macrophages.

Mounting evidence highlights a key role for IL-33 signaling in driving the stepwise progression of gastric cancer pathogenesis. Using a mouse model, we previously showed that IL-33 administration is sufficient to induce inflammation and mucous metaplasia in the gastric epithelium, via induction of Th2 cytokines, infiltration of myeloid cells and ILC2 cells. [7] Subsequent mouse genetic studies showed that development of premalignant gastric metaplasia is dependent on intact IL-33 signaling following parietal cell loss. Disruption of IL-33 signaling via loss of IL-33 ligand or its cognate ST2 receptor was sufficient to block both metaplasia, as well as the attendant M2 macrophage polarization that drives further oncogenic progression [46]. A recent study identified an IL-33/mast cell/M2 macrophage axis promoting gastric tumorigenesis, where genetic deficiency in either ST2, mast cells or *in vivo* depletion of macrophages all restricted gastric tumour growth in *gp130*^F/F^ mice. Importantly, the same study illustrated that mast cell numbers are elevated in human intestinal-type GC tissues and that high expression of an IL-33/mast cell activation gene signature predicts poor outcome in GC patients [32].

Our study supports and extends these studies, linking IL-33 signaling to gastric tumorigenesis; whereas Eissmann et al. investigated the impact of genetic deficiency in ST2, the cognate receptor for IL33 in GC, [32] we show a direct functional requirement for IL-33 as a driver of GC pathogenesis. What these studies collectively show is that genetic disruption of the IL-33 axis, either at the level of the IL-33 ligand production (gastric epithelium) or ST2 receptor activation on cellular targets (i.e., mast cells), is sufficient to interrupt gastric tumorigenesis. These provide strong evidence to support the IL-33/ST2 axis as an oncogenic driver of GC progression, that acts via unidirectional signaling between gastric epithelium and immune compartment to establish an immunosuppressive and pro-tumorigenic microenvironment.

Another important observation in our study comes from cell sorting studies showing that whilst gastric (and tumour) epithelial cells are the predominant source of IL-33 production in gastric tumours, tumour infiltrating (CD11b^+^ CD63^+^ CX3CR1^+^) macrophages were identified as a second significant source of this cytokine. As such, recruitment of macrophages downstream of mast cell activation may, via IL-33 production, additionally reinforce mast cell recruitment and overall protumorigenic cytokine responses. On the other hand, though clearly expressing IL-33, we found no evidence for presence of ST2 expression among tumour associated macrophages recovered by cell sorting. We also found evidence to support a role for IL-33 in promoting expansion of key mast cell subsets, including CTMC, which typically accumulate at tumour margins and are reactive to toluidine blue staining. Extending the study by Eissmann et al. which focused on CTMC, we also analysed the MMC subset, which have a predominant intratumoural distribution and, importantly, are not revealed by toluidine blue [41]. We too observed IL-33 dependent enrichment of toluidine blue-stained CTMCs specifically at tumour margins in *gp130*^F/F^ mice. We also found evidence for increased activation and expansion of the MMC subset, based on IL-33 dependent expression of selective markers, *Mcpt1* and *Mcpt2*, within *gp130*^F/F^ antral stomachs, and enrichment specifically within CD45+ cell fractions isolated from tumours by flow sorting. Our findings are broadly consistent with those of earlier studies showing that under normal mucosal homeostasis, overall mast cell numbers are low with equal proportions of MMC and CTMC, while during mucosal inflammation, MMC numbers increase disproportionately and can outnumber CTMC by a ratio of 5:1 [47].

Distinguishing between mast cell subtypes has key mechanistic significance, given that MMC, in particular, have been shown to promote gastrointestinal cancer progression by mobilizing protumorigenic CD11b+Gr1+ myeloid cells. In a recent study by Xu and colleagues, MMC numbers and MMC-specific protease expression were found to increase significantly in a colorectal cancer mouse model. Production of mMCP-1 after MMC activation was shown to not only drive recruitment of CD11b+Gr1+ cells, but also to stimulate their immunosuppressive activity to inhibit anti-tumour T cell responses. Furthermore, by blocking MMC activity, CD11b+Gr1+ infiltration was reduced, and colorectal cancer development was interrupted [43].

Intriguingly, whilst reduced tumour burden in compound mutant *gp130*^F/F^/IL33^−/−^ mice correlated with a decreased prevalence of mast cells and M2 macrophages, there was broadly no difference in expression of ILC2 markers relative to single mutant *gp130*^F/F^ or *Il33*^−/−^ mice. This observation should not rule out a role for ILC2 cells, as the ‘ILC2-related’ cytokines IL-9 and IL-4, potent inducers of MMC and CTMC respectively were upregulated in *gp130*^F/F^ tumours in IL-33 dependent fashion [44]. As a caveat, we cannot exclude other cellular sources of IL-9 expression including T cells of the T-helper type 9 (Th9) lineage, or possibly even MMC which have also been reported to produce IL-9. Collectively, our findings support activation of a mast cell/M2 (CD163-positive) macrophage axis as the most likely pathway through which IL-33 promotes gastric tumorigenesis.

In summary, we provide the first direct *in vivo* evidence supporting IL-33 as a cytokine driver of gastric tumour progression. We show that gastric epithelial derived IL-33, via unidirectional signaling to myeloid cell specific ST2 receptors sets up a protumorigenic inflammatory mucosal environment characterized by infiltration with M2 macrophages and mast cells. Our data provide further functional context to support elevated IL-33 expression as a causal factor in human GC progression. Our findings suggest selective targeting of IL-33 or it’s signaling components, either alone or in combination with other agents as a possible therapeutic strategy to restrain GC progression particularly in individuals presenting with established intramucosal adenocarcinoma and for whom therapeutic options are limited.

## MATERIALS AND METHODS

### Human tissues

Gastric mucosal tissue from *H. pylori* positive gastritis individuals and disease-free controls, premalignant adjacent-to-cancer tissues with intestinal metaplasia, and GCs were obtained as described [48–52].

### Mice

Gastric tissues from HKβ-promoter-driven GM-CSF transgenic mice (*Gmcsf*^Tg^, BALB/c background), [53] and *HKβ*^−*/*−^ mice (C57BL/6 background) [54] were gifted by Ian van Driel (Bio21 Molecular Science andBiotechnology Institute, Melbourne, Australia). The following mouse strains were on C57BL/6 background: *Tff1^−/−^*, [34] *gp130*^F/F^ [55] (gifted by Matthias Ernst, Olivia Newton John Cancer Research Institute, Melbourne, Australia), and *Il33*^−/−^ (purchased from the trans-NIH Knock-Out Mouse Project Repository (http://www.komp.org). Matching wild-type (WT) littermate controls were included as needed.

### Tissue preparation

Stomachs were harvested, pinned out, photographed for macroscopic morphometric analysis, and divided in two: one half was frozen in liquid nitrogen for RNA extraction, the other fixed in 4% paraformaldehyde in PBS and processed for histology. Paraffin sections were stained with H&E or Alcian blue periodic acid Schiff (AB-PAS) for detection of neutral and acidic mucins. Sections were also stained with Toluidine Blue for detection of mast cells. Spleens were also harvested, weighted, and processed in halves for histology and RNA extraction.

### Quantitative morphometry and cell counting analysis

Macroscopic morphometry was performed on images of whole stomachs: lesions were outlined with software drawing tool and measured using ImageJ software. Microscopic morphometry was performed on images of AB-PAS slides captured at 40× magnification as followed: the gastric mucosal area and length of muscularis mucosae were outlined with software drawing tool and measured using ImageJ software. Measurements were converted to millimeters after comparison with a calibrated graticule for both macroscopic and microscopic analysis. For cell counting analysis, stained cells were counted at 200× magnification, expressed as average number of stained cells per gland (Ki67), and as total number of stained cells per length of either antral or fundic muscularis mucosae (CD163, Toluidine Blue).

### Semiquantitative analysis of Inflammation and pathology

All scorings were performed in a blinded fashion. H&E stomachs were scored for polymorphonuclear (PMN) or mononuclear (MN) infiltration on a scale of 0 to 4 as previously described [51].

### Immunohistochemistry

Immunostaining was performed on paraffin slides of stomachs as described previously (7). Reagents used to detect staining were anti-Ki67 1:600, anti-CD163 1:400 (both rabbit anti-mouse antibodies from Abcam), biotinylated anti-rabbit secondary antibody, horseradish-peroxidase streptavidin (Vector Laboratories), 3’-diaminobenzidine (Sigma-Aldrich) and hematoxylin.

### Gene expression by qRT-PCR

Total RNA was extracted using TRIzol reagent (Life Technologies), DNase treated (DNA removal kit, Invitrogen) and reverse transcribed to cDNA by MMLV reverse transcriptase (Promega) with oligo(dT). qRT-PCR using SYBR green chemistry was performed with primers designed using primer3 tool (http://www.bioinformatics.nl/cgi-bin/primer3plus/primer3plus.cgi) (Supplementary Table 1). Relative gene expression was determined by normalizing to expression of mouse or human ribosomal protein (RP)L32 (internal reference genes) using 2^−ΔΔCt^ method. Full details were described previously [56].

### Flow cytometry and cell sorting

Gastric mucosal cells were isolated essentially as previously described, without Percoll gradient treatment [57]. Cell suspensions were stained for surface markers specific to epithelial cells and macrophages using the following monoclonal antibodies: rat anti-mouse E-cadherin (FITC, 1:100, clone #114420, R&D system); CD45 (Alexa Fluor 700, clone 30-F11, 1:500), CD11b (BV421, clone M1/70, 1:500), I-A/I-E (PE/Cy7, clone M5/114.15.2, 1:100); mouse anti-mouse CD64 (PE, clone X54-5/7.1, 1:100), CX3CR1 (APC, clone SA011F11, 1:200) (all from BioLegend). Dead cells were excluded by propidium iodide dye staining. Cells were sorted by flow cytometry into epithelial cells (E-cad^+^), macrophage enriched and depleted subsets using gating strategy as summarized (Figure 4D). Total RNA was extracted from each population using RNeasy Mini Kit (Qiagen), DNase treated, converted to cDNA and analyzed by qRT-PCR as described above.

### Statistical analysis

Data are shown as mean ± SEM. Statistical analysis was performed using Graphpad Prism software, with 1 or 2 tailed Student’s *t*-test for parametric data and Mann-Whitney *U* test for nonparametric data, as informed by appropriate normality testing.

## SUPPLEMENTARY MATERIALS



## Abbreviations

IL: interleukin
GC: gastric cancer
IM: intestinal metaplasia
HP: *Helicobacter pylori*
qRT-PCR: quantitative reverse transcription and polymerase chain reaction
Th: T helper type
M2: type-2 macrophage
WT: wild type
PMN: polymorphonuclear leukocyte
MN: mononuclear leukocyte
Mφ: macrophage
