# Supplementary material for: IL-33 promotes gastric tumour growth in concert with activation and recruitment of inflammatory myeloid cells
Source: Oncotarget. 2022 Jun 1;13:785–99. doi: 10.18632/oncotarget.28238 (PMC9159270; doi:10.18632/oncotarget.28238)
Supplement: Supplementary file 1 [file oncotarget-13-28238-s001.pdf]

IL-33 promotes gastric tumour growth in concert with activation and recruitment of inflammatory myeloid cells

SUPPLEMENTARY MATERIALS

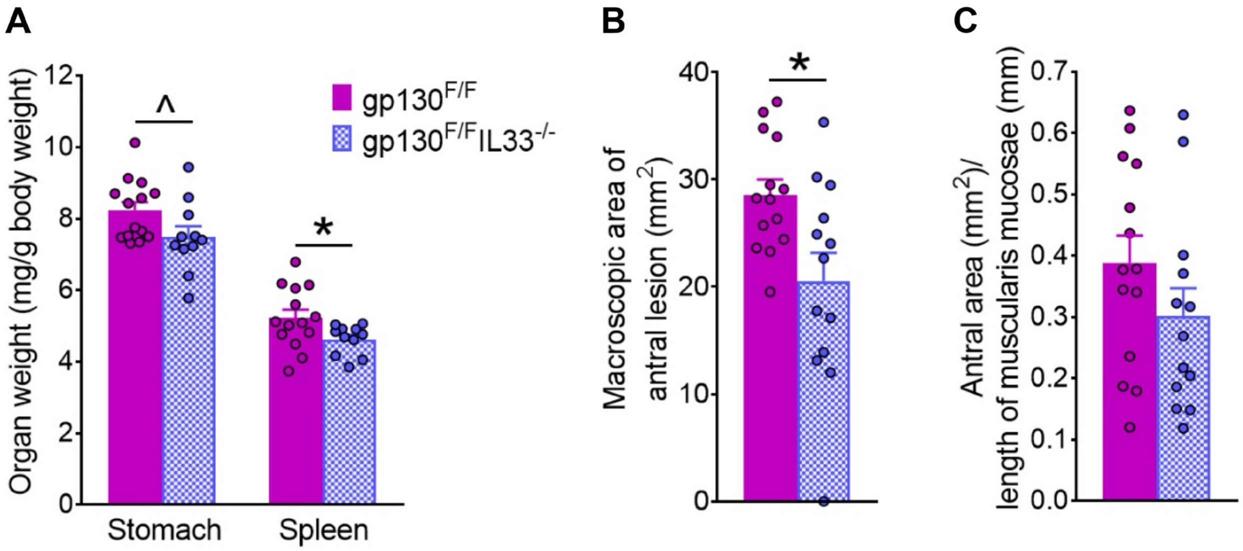

Supplementary Figure 1: Antral stomach tumorigenesis in 6 weeks old gp130$^{F/F}$ (*n* = 14) and gp130$^{F/F}$IL33$^{-/-}$ (*n* = 13) mice. (**A**) Stomach and spleen weight analysis. (**B**) Macroscopic morphometric analysis of antral lesions. (**C**) Microscopic morphometric analysis of antral mucosal thickness. One tailed student's *t* test: $^{\wedge}P < 0.05$. Two tailed student's *t* test: $^{*}P < 0.05$.

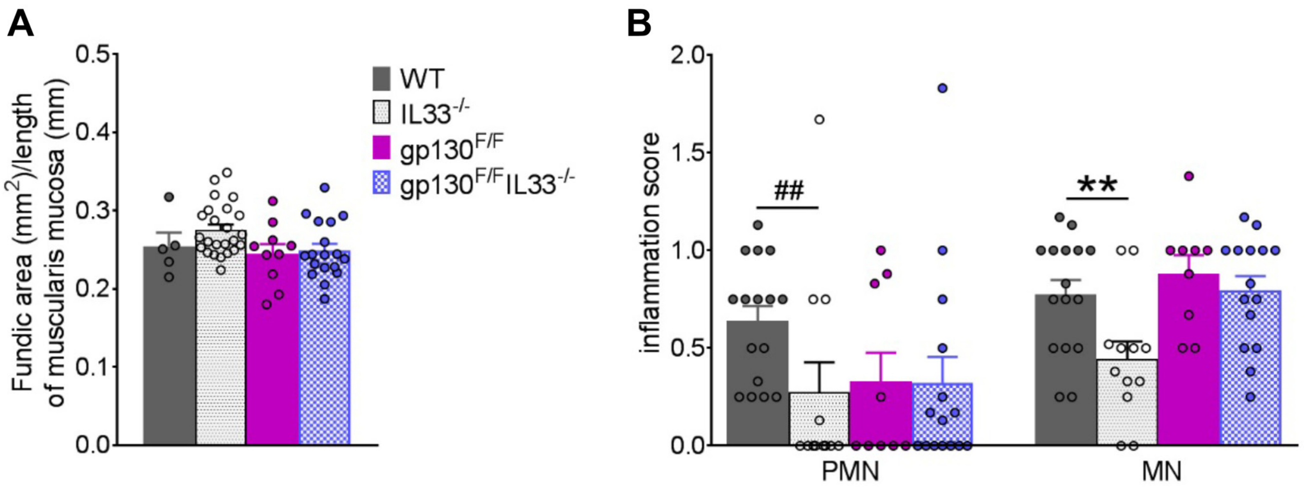

Supplementary Figure 2: Histological assessment of 12 weeks old fundus of WT, $IL33^{-/-}$, $gp130^{F/F}$ and $gp130^{F/F}IL33^{-/-}$ mice. (**A**) Microscopic morphometric analysis of fundic mucosal thickness. (**B**) Semiquantitative analysis of inflammation in the fundic mucosa. Abbreviations: PMN: polymorphonuclear cells; MN: mononuclear cells. WT (n = 16), $IL33^{-/-}$ (n = 12), $gp130^{F/F}$ (n = 9), $gp130^{F/F}IL33^{-/-}$ (n = 15). $^{**}P < 0.01$ (2 tailed Student's t test). $^{\#\#}P < 0.01$ (Mann Whitney test).

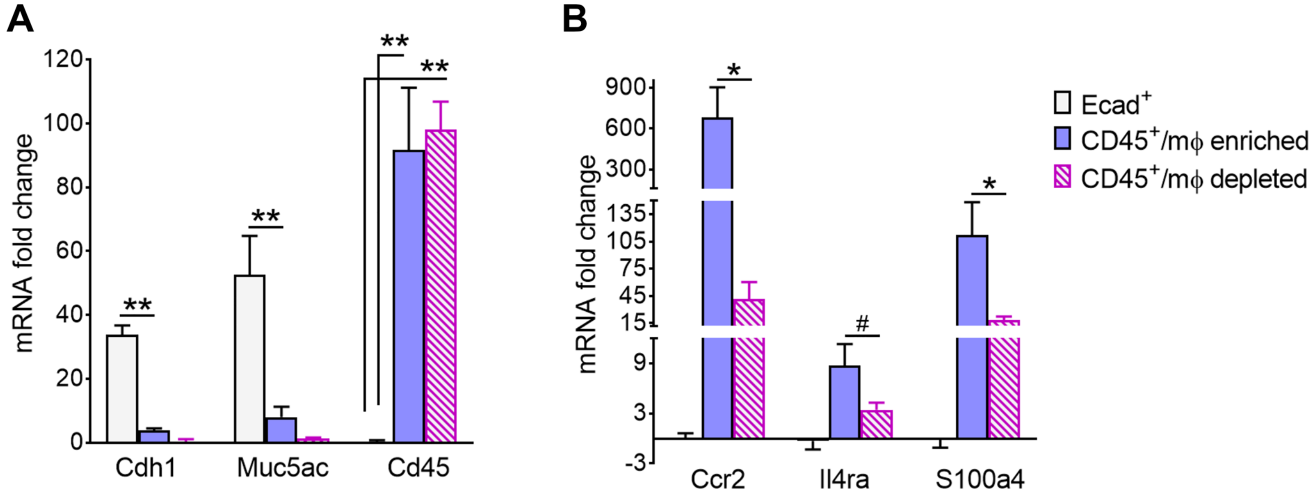

Supplementary Figure 3: Validation of flow cytometry sorted gastric mucosal cell populations in $gp130^{F/F}$ mice. Isolated populations: epithelial ($CD45^-$/$E\text{-}cad^+$), macrophage (Mϕ) enriched ($CD45^+$/$CD11b^+$/SSC^{low}/$CD64^+$/$CX3CR1^+$/$MHCII^+$), and Mϕ depleted ($CD45^+$/Mϕ depleted). (**A**, **B**) qRT-PCR analysis of isolated cell populations showing expression of (A) epithelial markers E-cadherin (Cdh1) and Muc5ac, as well as Cd45 marker; (B) Mϕ markers. Values are calculated relative to Mϕ depleted group for epithelial gene enrichment (Cdh1, Muc5ac), and relative to $Ecad^+$ group for immune gene enrichment (Cd45, Ccr2, Il4ra, S100a4). Student's t test: $^{*}P <$ 0.05, $^{**}P < 0.01$. Mann Whitney test: $^{\#}P < 0.05$.

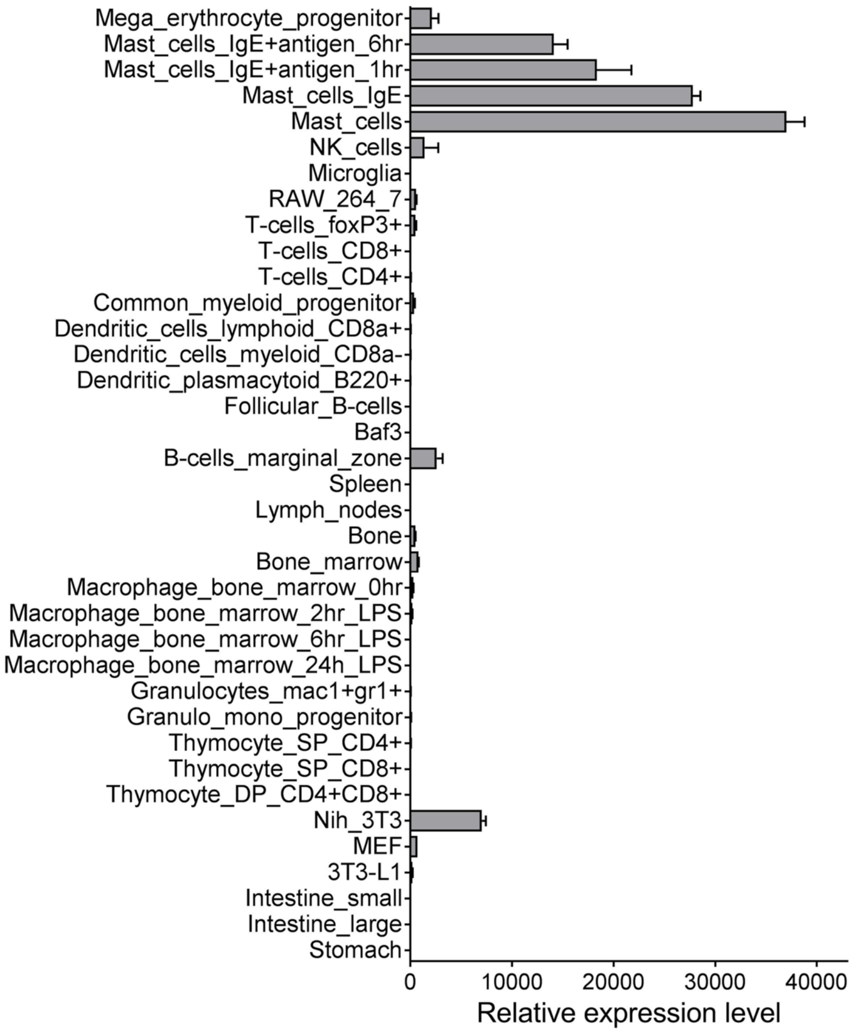

Supplementary Figure 4: Mast cell specific expression of St2 (Il1rl1). Microarray data showed relative expression of il1rl1 in various immune cell subsets. Source: Affymetrix MOE430_v2 probe set: 1425145_at (http://biogps.org/#goto=genereport&id=17082). Error bars show the SEM.

Supplementary Table 1: qRT-PCR primer list and sequences

Human primer sequences:

Gene	Forward Sequence	Reverse Sequence
RPL32	CATCTCCTTCTCGGCATCA	ACCCTGTTGTCAATGCCTC
IL33	TGTGGAGTGCTTTGCCTTTG	GGTGAAATTCCTGTGATACTTG
ST2	CGTGTAGAGCACTTTGTTCACC	CGCCTGCTCTTTCGTATGTTG
sST2	TCCCCTCTGTCTTTCAGTTTGG	AGATGAGTCACTGGCATACGAC

Mouse primer sequences:

Gene	Forward Sequence	Reverse Sequence
Areg	CTATCTTTGTCTGCCATCATC	CAACTTTTACCCTTGCAATTGCC
Arg1	AAAGCTGGTCTGTGGAAAA	ACAGACCGTGGGTTCTTCAC
CCR2	AAGTTCAGCTGCCTGCAAAGAC	GCCGTGGATGAACTGAGGTAAC
Cdh1	ACGTGGTAGACGTGAATGAAGC	CCGATACGTGATCTTCTGATCC
Cxcl2	AGT GAA CTG CGC TGT CAA TG	TTC AGG GTC AAG GCA AAC TT
Gata3	CGAGACATAGAGAGCTACGCAATC	ACCTGAGTAGCAAGGAGCGTAGA
IL10	TGATGCCCCAGGCAGAGA	CACCCAGGGAATTCAAATGC
IL11	CTGCAAGCCCGACTGGAA	AGGCCAGGCGAGACATCA
IL13	ATTGCATGGCCTCTGTAACC	GGCGAAACAGTTGCTTTGTG
IL17a	TCTGTGTCTCTGATGCTGTTGC	ACATTCTGGAGGAAGTCCTTGG
IL1a	AACCCATGATCTGGAAGAGACC	TGGTGCTGAGATAGTGTTTGTCC
IL1b	CAGGCAGTATCACTCATTGTGG	GTGCAGTTGTCTAATGGGAACG
IL22	TCGTCAACCGCACCTTTATG	GCCGGACATCTGTGTTGTTATC
IL33	GCTCACTGCAGGAAAGTACAGC	AACTTTGCCGGGGAAATCTTGG
IL4	GTCATCCTGCTCTTCTTTCTCG	TCACTCTCTGTGGTGTTCTTCG
IL4ra	GTGCCACATGGAAATGAATAGG	TGTGAGGTTGTCTGGAGCTAGG
IL5	AATGAGACGATGAGGCTTCCTG	CCCACGGACAGTTTGATTCTTC
IL6	ACAAAGCCAGAGTCCTTCAGAGA	CTGTTAGGAGAGCATTGGAAATTG
IL9	TCTTGCCTGTTTTCCATCGG	TCTGGTTGCATGGCTTTTCG
Mcpt1	TTGTGTTGACTGCTGCACAC	GTGTGGATTCACTCTTGCTCAC
Mcpt2	TTGTGATGACTGCTGCACAC	TATCTGCTGTGTGGGTTCGTTC
Muc5ac	GCAGTTGTGTCACCATCATCTGTG	GGGGCAGTCTTGACTAACCCTCTT
Rora	GCGGTTGACCTCGGCATAT	ACGCTGGACTCTGCTGTTACC
RpL32	5'GAGGTGCTGCTGATGTGC3'	5'GGCGTTGGGATTGGTGACT3'
sSt2	AGGTCGAAATGAAAGTTCCAGCA	CAAGCAATGTGTGAGGGACA
St2	AAGTGTTCTGGATTGAGGTTGC	GTAGAGCTTGCCATCGTTCC
Tgfb1	AATACAGGGCTTTCGATTCAGC	TAGTTGGTATCCAGGGCTCTCC
Tnfa	GTAGCCCACGTCGTAGCAAA	ACAAGGTACAACCCATCGGC
Ym1	TCTGGTGAAGGAAATGCGTAAA	GCAGCCTTGGAATGTCTTTCTC
